# Nuclear expression of p65 (RelA) in patients receiving post-operative radiotherapy for locally advanced squamous cell carcinoma of the head and neck

**DOI:** 10.1186/s12885-015-1107-2

**Published:** 2015-03-06

**Authors:** Dirk Rades, Stefan Huttenlocher, Nina D Seibold, Maximilian P Gebhard, Christoph Thorns, Katrin Hasselbacher, Barbara Wollenberg, Steven E Schild

**Affiliations:** 1 Department of Radiation Oncology, University of Lübeck, Ratzeburger Allee 160, D-23538 Lübeck, Germany; 2 Institute of Pathology, University of Lübeck, Lübeck, Germany; 3 Department of Oto-Rhino-Laryngology and Head and Neck Surgery, University of Lübeck, Lübeck, Germany; 4 Department of Radiation Oncology, Mayo Clinic Scottsdale, Scottsdale, AZ USA

**Keywords:** Head-and-neck cancer, Postoperative treatment, Nuclear factor kappa B, p65 (RelA), Outcomes

## Abstract

**Background:**

This study investigated the prognostic role of nuclear expression of p65 in patients with locally advanced squamous cell carcinoma of the head and neck (SCCHN) receiving post-operative radio(chemo)therapy.

**Methods:**

Nuclear p65-expression (H-score ≤50 versus >50) plus twelve characteristics were analyzed in 151 patients for overall survival (OS), metastases-free survival (MFS) and loco-regional control (LRC). Additional characteristics included age, gender, Karnofsky performance score (KPS), pre-radiotherapy hemoglobin levels, tumor site, histological grading, human papilloma virus (HPV)-status, T-classification, N-classification, American Joint Committee on Cancer (AJCC)-stage, extent of resection and concurrent chemotherapy. Univariate analyses were performed with Kaplan-Meier method and log-rank test, multivariate analyses with Cox proportional hazards model.

**Results:**

On univariate analyses, p65-expression had a significant impact on OS (p < 0.001) and LRC (p < 0.001) but not on MFS (p = 0.29). On multivariate analysis, KPS ≥80 (risk ratio [RR] 2.23; p = 0.012), HPV-positivity (RR 5.83; p = 0.020), T1-T2 (RR 1.38; p = 0.048), N0-N2a (RR 2.72; p = 0.005) and complete resection (RR 2.02; p = 0.049) were positively associated with OS; p65-negativity achieved borderline significance (RR 3.02; p = 0.052). Better MFS was associated with KPS ≥80 (RR 2.49; p = 0.015), T1-T2 (RR: 1.74; p = 0.005), N0-N2a (RR: 6.22; p < 0.001) and complete resection (RR 3.43; p = 0.003). Positive associations with LRC were found for p65-negativity (RR 5.06; p = 0.008), T1-T2 (RR: 1.49; p = 0.022), N0-N2a (RR: 2.97; p = 0.004) and favorable tumor site (RR 1.28; p = 0.025).

**Conclusions:**

P65-negativity was significantly associated with improved LRC and achieved borderline significance with respect to improved OS. Thus, p65-expression may be an additional target for novel agents in the treatment of locally advanced SCCHN.

## Background

Many patients with squamous cell carcinoma of the head and neck (SCCHN) present with locally advanced disease, which is often associated with unsatisfactory outcomes despite innovative treatment approaches [1-3]. Personalization of cancer treatment has become more popular during recent years and may provide beneficial effects also for the treatment of locally advanced SCCHN. The knowledge of significant prognostic factors, which characterize a cancer patient’s individual situation as precisely as possible, is important for individualized therapies. Several clinical and pre-clinical predictors have been identified for patients with locally advanced SCCHN [4-7]. Recently, the prognostic role of nuclear factor kappa B (NF-ĸB) has been suggested in a retrospective study of 101 SCCHN patients treated with definitive radio-chemotherapy [8]. In that study based on material from biopsies, nuclear expression of p65 (RelA), a transcription factor belonging to the NF-ĸB family, was associated with worse overall survival (OS) and metastases-free survival (MFS) in addition to a trend towards worse loco-regional control (LRC). In addition, Nakayama et al. found in their series of 36 untreated biopsy specimens from patients with SCC and 15 specimens with epithelial dysplasia of the oral cavity that high expression of p65 was associated with malignant behavior [9].

In several countries worldwide, the majority of patients with locally advanced SCCHN are treated with surgical resection of the primary tumor plus neck dissection followed by radiotherapy or, in case of specific risk factors, radio-chemotherapy instead of definitive radio-chemotherapy. Therefore, it would be important to know whether p65 (RelA) is also a significant predictor of treatment outcomes after adjuvant radio(chemo)therapy of locally advanced SCCHN. We feel that resected specimens provide more histologic material for successful staining than biopsies. Furthermore, in the previous clinical study, the human papilloma virus (HPV)-status and the hemoglobin levels prior to irradiation were not evaluated. Therefore, the current study was initiated. It investigated the potential prognostic role of p65 (RelA) in 151 patients treated with resection followed by radio(chemo)therapy for non-metastatic stage III/IV SCCHN.

## Methods

### Immunohistochemistry for p65 (RelA)

**Figure 1:**
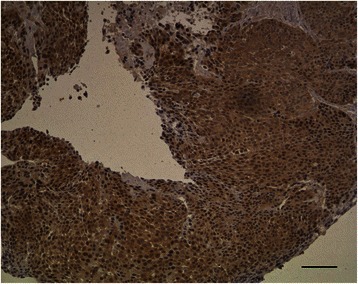
**Non-keratinizing carcinoma with immunostaining for p65: Brown staining of 30% nuclei in moderate intensity, original magnification 100x at microscope, bar = 100 μm.**

Nuclear expression of p65 (RelA) was analyzed in resected tumor specimens of 151 patients who had received obtained post-operative radiotherapy or radio-chemotherapy for locally advanced SCCHN. The study was approved by the local ethics committee (University of Lübeck). The patients have given written informed consent for the use of their data within retrospective studies in general. Children were not included in this study. Formalin-fixed and paraffin-embedded cancer tissues were sampled in a tissue micro array with cores of 2.0 mm (Manual Tissue Arrayer 1, Beecher Instruments, Silver Spring, MD, USA). Histological sections of 4 μm were de-paraffinized in xylene and rehydrated in graded alcohols. Antigene retrieval was processed according to standard operating procedure for our department of surgical pathology. Using 0.01 M citric acid pH6.0 for 5 min in a microwave at 850 W showed best results. Incubation with the primary antibody was done for 120 minutes in a dilution of 1:70. In a previous work by Balermpas et al., the polyclonal antibody ab31481 by Abcam plc, CB4 0FL Cambridge, UK) was used, but was not available anymore, when our study was performed [8]. After monoclonal antibody NF-kB p65 (clone L8F6) of Cell Signaling Technology Inc. (#6956) did not show any positive reaction, finally polyclonal rabbit Anti-NF-kB p65 antibody ab16502 (Abcam plc, CB4 0FL Cambridge, UK) was used. Visualization was performed with biotin-free poly-horseradish peroxidase-anti-mouse/rabbit/rat-IgG-kit (ImmunoLogic BV, 6921 VB Duiven, Netherlands). A diffuse large B-cell lymphoma served as positive control, and staining without primary antibody served as negative control. For differentiation between p65-negative and p65-positive tumors, the H-score was used [10]. The H-score was obtained as follows: % cells with weak staining × 1 plus % cells with moderate staining × 2 plus % cells with strong staining × 3. Tumors with an H-score of ≤50 were considered p65-negative and those tumors with an H-score of >50 were considered p65-positive. Microscopic evaluation was carried out by an experienced surgical pathologist, who was blinded to clinical and laboratory data. An example of a p65-positive tumor is given in Figure 1.

### Treatment

A total of 151 patients who had received surgery and postoperative radiotherapy or radio-chemotherapy for locally advanced SCCHN between 2000 and 2009 were included in this retrospective study. Further inclusion criteria were successful staining for p65, histologically proven SCC of the oropharynx, oral cavity, hypopharynx or larynx, and stage III or IV disease. Surgery was performed as resection of the primary tumor plus modified radical neck dissections. Conventionally fractionated radiotherapy (5 × 2 Gy per week) began 4–5 weeks after surgery. The PTV 1 included the primary tumor plus the bilateral cervical and supraclavicular lymph nodes (50–60 Gy). The PTV 2 included the primary tumor plus the involved lymph nodes; total doses were 60 Gy after complete resection and 66–70 Gy after incomplete resection. Lymph node levels with extra-capsular spread (ECS) received a total dose of 66 Gy. In case of incomplete resection and/or ECS, concurrent chemotherapy with 20 mg/m^2^ of cisplatin on days 1–5 and 29–33 plus/minus 600/1000 mg/m^2^ of 5-fluorouracil on days 1–5 and 29–33 was administered.

### Additional potential prognostic factors

Twelve variables were investigated in addition to nuclear expression of p65 (RelA) for associations with loco-regional control (LRC), metastases-free survival (MFS) and overall survival (OS). These twelve variables were age (≤60 years vs. ≥61 years), gender, Karnofsky performance score (KPS ≤70 vs. KPS ≥80), hemoglobin levels prior to irradiation (<12 g/dl vs. ≥12 g/dl), tumor site (oropharynx vs. hypopharynx vs. larynx vs. oral cavity/floor of mouth), histologic grade (G1-2 vs. G3), HPV-status of the tumor (negative vs. positive; HPV-positivity was defined as presence of both a specific p16-pattern and a positive in-situ hybridization-result.), T-classification (T1-T2 vs. T3-T4), N-classification (N0-N2a vs. N2b-N3), American Joint Committee on Cancer (AJCC) stage (stage III vs. stage IV) extent of resection (complete vs. incomplete) and administration of concurrent chemotherapy (no vs. yes). The distribution of these variables is given in Table 1.

**Table 1:** **Distribution of the potential prognostic variables**

	N patients (%)
**P65 expression**	
**Negative**	144 (95)
**Positive**	7 (5)
**Age**	
**≤ 60 years**	94 (62)
**≥ 61 years**	57 (38)
**Gender**	
**Female**	36 (24)
**Male**	115 (76)
**Karnofsky performance score**	
**≤ 70**	77 (51)
**≥ 80**	74 (49)
**Hemoglobin level pre-RT**	
**< 12 g/dl**	49 (32)
**≥ 12 g/dl**	102 (68)
**Tumor Site**	
**Oropharynx**	62 (41)
**Oral Cavity/Floor of mouth**	31 (21)
**Hypopharynx**	22 (15)
**Larynx**	36 (24)
**Histologic grade**	
**G1-2**	61 (40)
**G3**	90 (60)
**HPV status**	
**Negative**	129 (85)
**Positive**	19 (13)
**Not available**	3 (2)
**T-classification**	
**T1-T2**	71 (47)
**T3-T4**	80 (53)
**N-classification**	
**N0-N2a**	55 (36)
**N2b-N3**	96 (64)
**AJCC stage**	
**Stage III**	48 (32)
**Stage IV**	103 (68)
**Extent of resection**	
**Complete**	123 (81)
**Incomplete**	28 (19)
**Chemotherapy**	
**No**	82 (54)
**Yes**	69 (46)

### HPV-status

Formalin-fixed, paraffin-embedded tumor probes were analyzed for DNA of HPV-high-risk subtypes 16, 18, 31, 33 and 51 with in-situ-hybridization marked with anti-biotin-antibody processed in an autostainer. The morphologic criteria of positive hybridization-signals were adapted to the well-known morphology of CISH for other genes [11]. P16-immunoreactivity was qualitatively analyzed in accordance with morphologic criteria of intense confluent nuclear and cytoplasmic staining of tumor cells (positive). A tumor was defined as HPV-positive, if it showed both a specific p16-pattern as surrogate marker of oncogene activity of E6- and E7-gene and a positive in-situ hybridization-result. Positive control was a squamous-type carcinoma-in-situ of the cervix uteri of a patient with known HPV-subtype (HPV-Chip Type 3.5C, Chipron GmbH, Berlin, Germany). As negative control tumor tissue were stained with anti-biotin antibody only and cancer without p16-immunoreactivity and without amplification of HPV-high-risk-DNA in PCR with LCD-Array HPV-Chip 3.5 [12].

By chromogene in-situ-hybridization, we could identify DNA of human papilloma-virus high-risk subtypes 16, 18, 31, 33 and 51 in one assay but could not discriminate, which of the five subtypes was involved. In another assay we tested for low risk subtypes 6 and 11, but did not found them in carcinoma. If cases were positive for p16 in a cluster, which is typical for oncogene activation of HPV, but negative in chromogene in-situ-hybridization, we postulated another HPV-subtype than 16, 18, 31, 33 or 51 or a low sensitivity in paraffin embedded tissue, which was stored for years in an archive at room temperature.

### Statistical considerations

LRC, MFS and OS were referenced from the last day of radiotherapy/radio-chemotherapy. Univariate analyses were performed with the Kaplan-Meier method and the log-rank test. Those variables showing a significant or borderline significant (p ≤ 0.05) association with treatment outcomes on univariate analyses were also included in a multivariate analysis performed with the Cox proportional hazards model. If T-classification or N-classification and AJCC stage were significant in the univariate analysis, AJCC-stage was not included in the multivariate analysis, as it is a confounding variable of both T-classification and N-classification.

## Results

The median follow up was 21 months (range 0–67 months) in the entire cohort and 24 months (6–66 months) in those patients alive at the last follow up. Of those 19 patients with an HPV-positive tumor, 17 patients had oropharynx cancer, one patient larynx cancer and one patient cancer of the oral cavity.

On the univariate analysis of OS (Table 2), p65-negativity (Figure 1, p < 0.001), KPS ≥80 (p = 0.012), pre-radio(chemo)therapy hemoglobin levels of ≥12 g/dl (p = 0.002), HPV-positivity (p = 0.023), T-classification 1–2 (p = 0.009), N-classification 0-2a (p = 0.024), AJCC-stage III (p = 0.025) and complete resection (p = 0.017) were positively associated with survival. In the subsequent multivariate analysis (performed without AJCC-stage), KPS (risk ratio [RR] 2.36; 95%-confidence interval [CI] 1.26-4.48; p = 0.007), HPV-status (RR 5.51; 95%-CI 1.17-100.00; p = 0.027), T-classification (RR 1.39; 95%-CI 1.01-1.94; p = 0.041) and N-classification (RR 2.63; 95%-CI 1.29-5.90; p = 0.007) maintained significance. P65-expression achieved borderline significance (RR 3.02; 95%-CI 0.99-7.60; p = 0.052). Hemoglobin levels showed a trend (RR 1.76; 95%-CI 0.94-3.26; p = 0.08), and extent of resection was not significant (RR 1.81; 95%-CI 0.88-3.52; p = 0.11).

**Table 2:** **Univariate analysis of overall survival**

	At 1 year (%)	At 2 years (%)	At 3 years (%)	P
**P65 expression**				
**Negative**	90	71	68	
**Positive**	71	n.a.	n.a.	**<0.001**
**Age**				
**≤ 60 years**	89	68	66	
**≥ 61 years**	89	70	66	0.92
**Gender**				
**Female**	91	84	78	
**Male**	88	64	62	0.19
**Karnofsky performance score**				
**≤ 70**	88	57	49	
**≥ 80**	90	76	76	**0.012**
**Hemoglobin level pre-RT**				
**< 12 g/dl**	83	46	46	
**≥ 12 g/dl**	92	78	74	**0.002**
**Tumor Site**				
**Oropharynx**	93	76	72	
**Oral Cavity/Floor of mouth**	90	53	47	
**Hypopharynx**	77	56	56	
**Larynx**	89	75	75	0.35
**Histologic grade**				
**G1-2**	95	73	69	
**G3**	85	65	63	0.26
**HPV status**				
**Negative**	87	65	62	
**Positive**	100	93	93	**0.023**
**T-classification**				
**T1-T2**	94	82	77	
**T3-T4**	84	56	56	**0.009**
**N-classification**				
**N0-N2a**	96	81	81	
**N2b-N3**	85	61	57	**0.024**
**AJCC stage**				
**Stage III**	98	82	82	
**Stage IV**	85	62	58	**0.025**
**Extent of resection**				
**Complete**	91	73	70	
**Incomplete**	82	50	50	**0.017**
**Chemotherapy**				
**No**	88	69	67	
**Yes**	90	68	65	0.99

n.a. = not available; significant p-values are given in bold.

**Figure 2:**
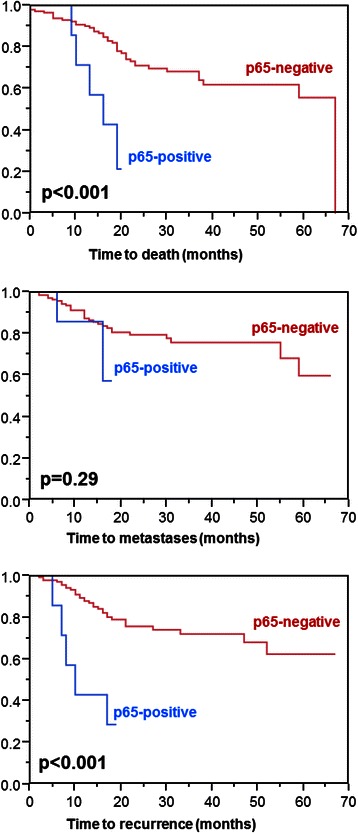
**Impact of p65-expression on overall survival (top), metastases-free survival (middle) and loco-regional control (bottom).**

On univariate analyses of MFS (Table 3), a positive impact was found for KPS ≥80 (p = 0.049), favorable tumor site (oropharynx or larynx cancer; p = 0.036), T-classification 1–2 (p = 0.013), N-classification 0-2a (p = 0.004), AJCC-stage III (p = 0.011) and complete resection (p = 0.002). HPV-positivity showed a trend towards improved MFS (p = 0.09). P65-expression was not significantly associated with MFS (Figure 2, p = 0.29). In the corresponding multivariate analysis (performed without AJCC-stage), KPS (RR 2.49; 95%-CI 1.19-5.29; p = 0.015), T-classification (RR: 1.74; 95%-CI: 1.18-2.64; p = 0.005), N-classification (RR: 6.22; 95%-CI: 2.34-21.56; p < 0.001) and extent of resection (RR 3.43; 95%-CI 1.54-7.34; p = 0.003) were significant; tumor site (RR: 1.13; 95%-CI: 0.88-1.43; p = 0.34) was not significant.

**Table 3:** **Univariate analysis of metastases-free survival**

	At 1 year (%)	At 2 years (%)	At 3 years (%)	P
**P65 expression**				
**Negative**	87	79	76	
**Positive**	86	n.a.	n.a.	0.29
**Age**				
**≤ 60 years**	89	77	74	
**≥ 61 years**	85	82	76	0.90
**Gender**				
**Female**	91	84	77	
**Male**	86	77	75	0.41
**Karnofsky performance score**				
**≤ 70**	82	72	63	
**≥ 80**	91	83	81	**0.049**
**Hemoglobin level pre-RT**				
**< 12 g/dl**	83	75	75	
**≥ 12 g/dl**	89	80	75	0.79
**Tumor Site**				
**Oropharynx**	93	87	81	
**Oral Cavity/Floor of mouth**	82	70	70	
**Hypopharynx**	72	55	55	
**Larynx**	91	87	79	**0.036**
**Histologic grade**				
**G1-2**	86	78	73	
**G3**	88	79	76	0.90
**HPV status**				
**Negative**	87	76	72	
**Positive**	95	95	95	0.09
**T-classification**				
**T1-T2**	95	88	85	
**T3-T4**	80	71	65	**0.013**
**N-classification**				
**N0-N2a**	96	90	90	
**N2b-N3**	82	72	67	**0.004**
**AJCC stage**				
**Stage III**	95	89	89	
**Stage IV**	83	73	68	**0.011**
**Extent of resection**				
**Complete**	90	84	80	
**Incomplete**	74	57	57	**0.002**
**Chemotherapy**				
**No**	88	86	82	
**Yes**	86	71	68	0.16

n.a. = not available; significant p-values are given in bold.

The univariate analysis of LRC (Table 4) revealed positive associations with LRC for p65-negativity (Figure 2, p < 0.001), pre-radio(chemo)therapy hemoglobin levels of ≥12 g/dl (p = 0.037), HPV-positivity (p = 0.041), T-classification 1–2 (p = 0.007), N-classification 0-2a (p = 0.040) and AJCC-stage III (p = 0.042). Favorable tumor sites achieved borderline significance (p = 0.050). In the multivariate analysis (performed without AJCC-stage), p65-expression (RR 5.06; 95%-CI 1.63-13.24; p = 0.008), T-classification (RR: 1.45; 95%-CI: 1.03-2.09; p = 0.033), N-classification (RR: 2.83; 95%-CI: 1.31-6.81; p = 0.007) and tumor site (RR 1.36; 95%-CI 1.09-1.71; p = 0.008) remained significant. Hemoglobin levels (RR 1.64; 95%-CI 0.82-3.17; p = 0.16) and HPV-stage (RR 2.49; 95%-CI 0.49-45.45; p = 0.32) were not significantly associated with LRC.

**Table 4:** **Univariate analysis of loco-regional control**

	At 1 year (%)	At 2 years (%)	At 3 years (%)	P
**P65 expression**				
**Negative**	88	76	72	
**Positive**	43	n.a.	n.a.	**<0.001**
**Age**				
**≤ 60 years**	83	70	67	
**≥ 61 years**	90	79	75	0.23
**Gender**				
**Female**	91	78	78	
**Male**	84	71	67	0.31
**Karnofsky performance score**				
**≤ 70**	83	69	64	
**≥ 80**	88	77	74	0.19
**Hemoglobin level pre-RT**				
**< 12 g/dl**	79	60	60	
**≥ 12 g/dl**	89	78	73	**0.037**
**Tumor Site**				
**Oropharynx**	91	83	79	
**Oral Cavity/Floor of mouth**	74	46	46	
**Hypopharynx**	82	71	71	
**Larynx**	87	77	69	**0.050**
**Histologic grade**				
**G1-2**	91	78	74	
**G3**	82	70	67	0.24
**HPV status**				
**Negative**	84	69	65	
**Positive**	94	94	94	**0.041**
**T-classification**				
**T1-T2**	92	83	77	
**T3-T4**	80	64	64	**0.007**
**N-classification**				
**N0-N2a**	90	82	82	
**N2b-N3**	83	68	63	**0.040**
**AJCC stage**				
**Stage III**	91	82	82	
**Stage IV**	83	68	63	0.042
**Extent of resection**				
**Complete**	88	75	71	
**Incomplete**	78	67	67	0.46
**Chemotherapy**				
**No**	87	77	70	
**Yes**	85	69	69	0.99

n.a. = not available; significant p-values are given in bold.

## Discussion

The outcomes after treatment of patients with locally advanced SCCHN need to be improved. In recent years, promising new molecular targeted therapies have been introduced for these patients including epidermal growth factor receptor monoclonal antibodies, tyrosine kinase inhibitors, vascular endothelial growth factor receptor inhibitors, and inhibitors of other pathways and targets [13]. Furthermore, modern radiation techniques have been more widely used for the treatment of SCCHN including intensity-modulated radiotherapy, adaptive intensity-modulated radiotherapy and proton therapy, either alone or in combination with systemic agents [14-16].

A retrospective study published in 2010 suggested the expression of NF-ĸB to be a new marker for decreased radio-sensitivity in patients irradiated for early-stage cancer of the larynx [17]. The study matched 35 patients with a local recurrence to 70 patients without a recurrent tumor. NF-ĸB was significantly associated with local failure in both the univariate (p = 0.008) and the multivariate (p = 0.04) analysis. In recurrent cancers, expression of NF-ĸB was markedly increased in 80% (23 of 29) of tumors. In 2013, another retrospective study of 77 patients receiving radiotherapy or radio-chemotherapy for SCCHN with a relatively favorable prognosis (64% T1-2, 70% N0, 48% AJCC-stage I-II) was reported [18]. Local control at 5 years was significantly better with low expression of NF-ĸB than with higher expression of NF-ĸB (80% vs. 42%, p = 0.001). Also this study suggested a correlation between high levels of expression of NF-ĸB and decreased radio(chemo)-sensitivity. These data were supported by another retrospective study of 101 patients treated with radio-chemotherapy for locally advanced SCCHN [8]. Mean follow up time was 25 months (range 2.3-63 months). Patients with a p65-positive tumor had a worse prognosis than those patients with a p65-negative tumor. The two-year OS rates were 40% and 67%, respectively (p = 0.008), the 2-year MFS rates 38% and 62%, respectively (p = 0.008), and the 2-year LRC rates 40% and 62%, respectively (p = 0.069). These three retrospective studies were performed in patients treated with definitive radiotherapy or radio-chemotherapy [8,17,18]. In contrast, the present study was performed in patients receiving radio(chemo)therapy in an adjuvant setting following resection of the primary tumor plus neck dissection. Furthermore, two of the previous studies were performed in SCCHN patients with a more favorable prognosis (48%-100% early stage tumors). Therefore, the comparison of our study to the three previous studies has some limitations.

In accordance with the three previous studies [8,17,18], our study also revealed a significant impact of the nuclear expression of NF-ĸB (p65/RelA) on treatment outcomes. The prognostic role of the nuclear expression of NF-ĸB (p65/RelA) may form the basis for the development of new targeted therapies for locally advanced SCCHN. However, the retrospective design of the four studies creates the risk of including inapparent selection biases. We aimed to reduce the risk of such biases by including only patients who had received resection plus neck dissection followed by radio(chemo)therapy. The cancer specimens were from resection and not from biopsy, which may have reduced a potential influence of the quality of staining for p65. In general, staining will provide better results if it is performed in larger resected specimens rather than in smaller specimens from biopsies. However, these results of our study and of the three previous reports from the literature should ideally be confirmed prospective cohorts of patients with locally advanced SCCHN [8,17,18].

Improved treatment outcomes were also significantly associated with better performance status (KPS ≥80), favorable tumor site (oropharynx or larynx), HPV-positivity, lower tumor stage (T1-2, N0-2a, AJCC-stage III) and complete resection in the present study. Hemoglobin levels prior to radiotherapy of ≥12 g/dl showed a trend towards better OS. These findings agree with the data from the reported literature which reveals consistency of our results [19-23].

## Conclusions

P65-negativity was significantly associated with improved LRC and achieved borderline significance with respect to improved OS. Thus, p65-expression may be an additional target for novel agents in the treatment of locally advanced SCCHN.
